# Supplementary material for: Genetic Profile of Patients with Limb-Girdle Muscle Weakness in the Chilean Population
Source: Genes (Basel). 2022 Jun 16;13(6):1076. doi: 10.3390/genes13061076 (PMC9223019; doi:10.3390/genes13061076)
Supplement: Supplementary file 1 [file genes-13-01076-s001.zip › genes-1739915-supplementary/Supplementary Material/Supplementary File S1.pdf]

Supplementary File S1. Genes Included in the Different NGS Panels

1. **MyoPanel2.** 306 genes panel, Marseille, France: AAARS, ABCC9, ABHD5, ACADVL, ACTA1, ACTC1, ACTN2, ACVR1, AGL, AGRN, ALDH3A2, ALS2, ANG, ANK2, ANKRD1, ANO5, APTX, AR, ARHGEF10, ATL1, ATM, ATP2A1, ATP7A, ATXN1, ATXN10, ATXN2, ATXN3, ATXN7, BAG3, BEAN1, BIN1, BSCL2, C10ORF2, CABC1, CACNA1A, CACNA1C, CACNA1S, CACNB2, CACNB4, CAPN3, CASQ2, CAV3, CFL2, CHAT, CHKB, CHRNA1, CHRNB1, CHRND, CHRNE, CHRNG, CLCN1, CNBP, CNTN1, COL6A1, COL6A2, COL6A3, COLQ, COX15, CPT2, CRYAB, CSRP3, CTDP1, CYP7B1, DAG1, DCTN1, DES, DMD, DMPK, DNAJB6, DNM2, DNMT1, DOK7, DSC2, DSG2, DSP, DTNA, DUX4, DUX4, DYNC1H1, DYSF, EGR2, EMD, ENO3, ERBB3, ETFA, ETFB, ETFDH, EYA4, FA2H, FBLN5, FGD4, FGF14, FHL1, FIG4, FKRP, FKTN, FLNA, FLNC, FUS, FXN, GAA, GAN, GARS, GBE1, GDAP1, GFPT1, GJA5, GJB1, GLE1, GNE, GPD1L, GYG1, GYS1, HCN4, HOXD10, HSPB1, HSPB3, HSPB8, HSPD1, HSPG2, IFRD1, IGHMBP2, IKBKAP, ILK, ISCU, ITGA7, ITPR1, JPH2, JUP, KBTBD13, KCNA1, KCNA5, KCNC3, KCNE1, KCNE2, KCNE3, KCNH2, KCNJ18, KCNJ2, KCNQ1, KIAA0196, KIAA0415, KIF1A, KIF1B, KIF21A, KIF5A, KLHL9, L1CAM, LAMA2, LAMA4, LAMB2, LAMP2, LARGE, LDB3, LDHA, LITAF, LMNA, LPIN1, MATR3, MED25, MFN2, MPZ, MRE11A, MRPL3, MSTN, MTM1, MTMR2, MTTP, MURC, MUSK, MYBPC3, MYH2, MYH3, MYH6, MYH7, MYH8, MYL2, MYL3, MYLK2, MYOT, MYOZ2, MYPN, NDRG1, NDUFAF1, NEB, NEFL, NEXN, NGF, NIPA1, NPPA, OPA1, PABPN1, PEX7, PFKM, PGAM2, PGK1, PGM1, PHKA1, PHOX2A, PHYH, PIP5K1C, PKP2, PLEC, PLEKHG5, PLN, PLP1, PMP22, PNPLA2, PNPLA6, POLG, POLG2, POMGNT1, POMT1, POMT2, PPP2R2B, PRKAG2, PRKCG, PRPS1, PRX, PSEN2, PTRF, PYGM, RAB7A, RAPSN, RBM20, REEP1, RRM2B, RYR1, RYR2, SACS, SBF2, SCN4A, SCN5A, SEPN1, SEPT9, SETX, SGCA, SGCB, SGCD, SGCE, SGCG, SH3TC2, SIL1, SLC12A6, SLC1A3, SLC22A5, SLC25A20, SLC25A4, SLC33A1, SMN1, SOD1, SPAST, SPG11, SPG20, SPG21, SPG7, SPTBN2, SPTLC1, SPTLC2, SUCLA2, SYNE1, SYNE2, TARDBP, TAZ, TBP, TCAP, TDP1, TGFB3, TK2, TMEM43, TMPO, TNNC1, TNNI2, TNNI3, TNNT1, TNNT2, TNNT3, TOR1A, TPM1, TPM2, TPM3, TRIM32, TRPV4, TTBK2, TTN, TTPA, TTR, TUBB3, VAPB, VCL, VCP, VRK1, WNK1, YARS, ZFYVE26, ZFYVE27
2. **DLE-NGS – DLE Laboratory, Sao Paulo, Brazil (Sanofi-Genzyme)** 10 genes panel, 9 most frequent recessive LGMD and Pompe disease, DLE Labs, Sao Paulo, Brazil: CAPN3; DYSF; SGCG; SGCA; SGCB; SGCD; FKRP; ANO5; TCAP, GAA.
3. **CL-NGS,** ICBM locally designed 15 LGMD gene panel: LMNA; CAV3; DNAJB6; CAPN3; DYSF; SGCG; SGCA; SGCB; SGCD; FKRP; ANO5; FKTN; EMD; FHL1; DES.
4. **Invitae™ Comprehensive Neuromuscular Disorders Panel (CNMDP)**. 123 gene Comprehensive Neuromuscular Panel, California, USA: ACTA1, AGRN, ALG14, ALG2, ANO5, ATP2A1, B3GALNT2, B4GAT1, BAG3, BIN1, CACNA1S, CAPN3, CAV3, CCDC78, CFL2, CHAT, CHKB, CHRNA1, CHRNB1, CHRND, CHRNE, CLCN1, CNTN1, COL12A1, COL6A1, COL6A2, COL6A3, COLQ, CPT2, CRYAB, DAG1, DES, DMD, DNAJB6, DNM2, DOK7, DPAGT1, DPM1, DPM2, DPM3, DYSF, EMD, FHL1, FKBP14, FKRP, FKTN, FLNC, GAA, GFPT1, GMPPB, GNE, HNRNPA2B1, HNRNPDL, ISPD, ITGA7, KBTBD13, KCNJ2, KLHL40, KLHL41, LAMA2, LAMB2, LAMP2, LARGE1, LDB3, LIMS2, LMNA, LMOD3, LRP4, MATR3, MEGF10, MTM1, MUSK, MYF6, MYH2, MYH7, MYL2, MYOT, MYPN, NEB, PLEC, PNPLA2, POMGNT1, POMGNT2, POMK, POMT1, POMT2, PREPL, RAPSN, RYR1, SCN4A, SELENON, SGCA, SGCB, SGCD, SGCG, SMCHD1, SMN1, SMN2, SNAP25, SQSTM1, STAC3, STIM1, SUN1, SUN2, SYNE1, SYNE2, TAZ, TCAP, TIA1, TMEM43, TMEM5, TNNT1, TNPO3, TOR1AIP1, TPM2, TPM3, TRAPPC11, TRIM32, TTN, VCP, VMA21.

In patient P53-Myo120, a 137 gene panel was performed. It adds to the 123-gene panel the following genes: ADSSL1, AMPD1, CASQ1, COL13A1, GOSR2, GYG1, GYS1, HACD1, ISCU, MAP3K20, MICU1, MYO18B, ORAI1, PYROXD1, RXYLT1, SLC18A3, SLC5A7, SPEG, SYT2, TK2, VAMP1, and excludes the following genes: LAMB2, LRP4, MYF6, SNAP25, TMEM5
