# Supplementary material for: Genetic Profile of Patients with Limb-Girdle Muscle Weakness in the Chilean Population
Source: Genes (Basel). 2022 Jun 16;13(6):1076. doi: 10.3390/genes13061076 (PMC9223019; doi:10.3390/genes13061076)
Supplement: Supplementary file 1 [file genes-13-01076-s001.zip › genes-1739915-supplementary/Supplementary Material/Supplementary Table S1.pdf]

Supplementary Table S1. Summary of the clinical and genetic screening of the Chilean series of patients with LGMW

ID	Local ID	Suspected Diagnosis	Other diagnostic tests	Definite Diagnosis	Screening Method	Affected Gen	Pathogenic or most probably potentially pathogenic variants found	Genomic Position (hg19)	ACMG codes	ACMG classification
P1	Myo004	LGMDR1	Bx, MRI, lab, EcoK, Sp. EMG, SEG	LGMDR1-Calpain 3 related myopathy	MyoPanel2[1]	*CAPN3*	Ex22: NM_000070.3:c.2362_2363delinsTCATCT; p.(Arg788SerfsTer14) HOZ	15:42703180-42703181	PVS1, PM3_very_strong, PM2, PP4_mod	Pathogenic
P2	Myo004.1	LGMDR1	Bx, MRI, lab, EcoK, Sp. EMG, SEG	LGMDR1-Calpain 3 related myopathy	MyoPanel2	*CAPN3*	Ex22: NM_000070.3: c.2362_2363delinsTCATCT; p.(Arg788SerfsTer14) HOZ	15:42703180-42703181	PVS1, PM3_very_strong, PM2, PP4_mod	Pathogenic
P3	Myo017	hiCK, arrhythmia	Bx, MRI, lab, EcoK, Sp. EMG, SEG	LGMDR12-Anoctamin 5 related myopathy	MyoPanel2	*ANO5*	Ex19: NM_213599.3:c.2201T>C; p.(Leu734Pro) HOZ	11:22294501	PM2, PM3, PP3, PP4_mod	Likely pathogenic
P4	Myo023	hiCK	Bx, MRI, lab, EcoK, Sp. EMG	Congenital Paramyotonia	MyoPanel2	*SCN4A*	Ex22: NM_000334.4: c.3938C>T; p.(Thr1313Met) HEZ	17:62021185	PS3, PS4, PM2, PP3, PP4_mod	Pathogenic
P5	Myo029	Myalgia, hiCK	Bx, lab, EMG	*RYR1*-related myopathy?	MyoPanel2	*RYR1*	Ex6: NM_000540.3: c.487C>T; p.(Arg163Cys) HEZ	19:38934851	PS3, PS4, PM2, PP3, PP4	Pathogenic
						MYH7	Ex20: NM_000257.4:c.2167C>T; (p.Arg723Cys) HEZ	14:23895023	PS4, PP1_ Strong, PM1, PM2, PM5, PM6, PP3	Pathogenic
P6	Myo031	LGMW, hiCK,	Bx, lab, EcoK, Sp. EMG	Compatible with titinopathy	CL-NGS[2]; MyoPanel2	*TTN*	Ex340: NM_001267550.2:c.94507G>A:p.(Ala31503Thr) HEZ	2:179411745	PP3, PP4	VUS
P7	Myo042	Dysferlinop.	Lab, EcoK, Sp. EMG	LGMDR2-Dysferlin related myopathy	DLE-NGS[3]	*DYSF*	Ex6: NM_003494.4:c.526C>T; p.(Gln208Ter) HEZ	2: 71740914	PVS1, PM3, PM2, PP4_mod	Pathogenic
							Ex13: NM_003494.4: c.1276G>A; p.(Gly426Arg) HEZ	2:71755523	PM3_strong, PM2, PP3, PP4_mod	Likely pathogenic
P8	Myo050	LGMDR1	Bx, MRI, lab, EcoK, Sp. EMG, SEG	LGMDR1-Calpain 3 related myopathy	MyoPanel2	*CAPN3*	Ex1: NM_000070.3:c.107delG: p.(Gly36ValfsTer21) HOZ	15:42652105	PVS1, PM2, PM3, PP4_mod	Pathogenic
P9	Myo052	LGMDR, hi-CK	Bx, MRI, lab, EcoK, Sp. EMG	LGMDR9-FKRP-related myopathy	MyoPanel2	*FKRP*	FKRP: Ex4: NM_024301.5:c.919T>G; p.(Tyr307Asp) HEZ	19:47259626	PM5, PM3_sup, PM2, PP3, PP4_mod	Likely pathogenic
							FKRP: Ex4: NM_024301.5:c.877A>C; p.(Thr293Pro) HEZ	19:47259584	PM5, PM3_sup, PM2, PP3, PP4_mod	Likely pathogenic
P10	Myo053	LGMD-R3-6	Bx, MRI, lab, EcoK, Sp. EMG, Ab	IMNM Anti HMGCR-Ab	CL-NGS; MyoPanel2	*None*				
P11	Myo054	LGMD-R?	Bx, MRI, lab, EcoK, Sp. EMG	Unknown	MyoPanel2	*None*				
P12	Myo055	LGMD-R?	Bx, MRI, lab, EcoK, Sp. EMG	Unknown	MyoPanel2	*None*				
P13	Myo056	LGMD-R?	Bx, MRI, lab, EcoK, Sp. EMG, Ab	IMNM Anti-SRP Ab. Deceased esophageal hemorrhage	CL-NGS, DLE-NGS	*None*				
P14	Myo057	LGMDR-3-6	Bx, MRI, lab, EcoK, Sp. EMG, SEG	LGMDR1-Calpain 3 related myopathy	MyoPanel2	*CAPN3*	Ex1: NM_000070.3:c.107delG p.(Gly36ValfsTer21) HEZ	15:42652105	PVS1, PM2, PM3, PP4_mod	Pathogenic
							Ex21: NM_000070.3:c.2243G>A; p.(Arg748Gln) HEZ	15:42702844	PM3_VS, PS3_sup, PM1, PM2, PP3, PP1_mod; PP4_mod	Pathogenic

Supplementary Table S1. Summary of the clinical and genetic screening of the Chilean series of patients with LGMW (Continued)

ID	Local ID	Suspected Diagnosis	Other diagnostic tests	Definite Diagnosis	Screening Method	Affected Gen	Pathogenic or most probably potentially pathogenic variants found	Genomic Position (hg19)	ACMG codes	ACMG classification
P15	Myo058	LGMDR-3-6	Bx, MRI, lab, EcoK, Sp. EMG	Unknown	DLE-NGS; CL-NGS; MyoPanel2, CNMDP(4a)	*None*				
P16	Myo064	LGMD-R?	Bx, MRI, lab, EcoK, Sp. EMG, Ab	IMNM Anti HMGCR-Ab	DLE-NGS; CL-NGS; MyoPanel2	*None*				
P17	Myo067	LGMD-R3-6	Bx, MRI, lab, EcoK, Sp. EMG	LGMD R5 γ-Sarcoglycan related myopathy. SGCG absent in biopsy	DLE-NGS, CL-NGS, MyoPanel2, CNMDP	*SGCG*	Deletion entire code sequence		PSV1_strong, PM4, PP4_mod	Pathogenic
P18	Myo068	LGMW	Bx, MRI, lab, EcoK, Sp. EMG	LGMD R1-Calpain 3 related myopathy	DLE-NGS; MyoPanel2	*CAPN3*	Ex10: NM_000070.3: c.1333G>C; p.(Gly445Arg); HEZ	15:42691829	PM3_strong, PS3, PM1, PM2, PP3	Pathogenic
							Ex22: NM_000070.3: c.2362_2363delinsTCATCT; p.(Arg788SerfsTer14) HEZ	15:42703180-42703181	PVS1, PM3_very_strong, PM2	Pathogenic
P19	Myo074	Dysferlinop.	Bx, MRI, lab, EcoK, Sp. EMG	LGMDR2-Dysferlin related myopathy	NGS-DLE	*DYSF*	Ex27: NM_003494.4:c.2858dupT; p.(Phe954ValfsTer2) HOZ	2:71796997	PVS1, PM2, PM3, PM4, PP4_mod	Pathogenic
P20	Myo075	hiCK, myalgia	Bx, MRI, lab, EcoK, Sp. EMG, Ab.	Fulfil diagnostic criteria for ALS	DLE-NGS, CL-NGS, CNMDP	*None*				
P21	Myo078	Dysferlinop.	Bx, MRI, lab, EcoK, Sp. EMG, Ab.	LGMD R2-Dysferlin related myopathy	NGS-DLE	*DYSF*	Ex27: NM_003494.4:c.2858dupT; p.(Phe954ValfsTer2) HOZ	2:71796997	PVS1, PM2, PM3, PM4, PP4_mod	Pathogenic
P22	Myo079	Dysferlinop.	Bx, MRI, lab, EcoK, Sp. EMG, Ab.	LGMD R2-Dysferlin related myopathy	NGS-DLE	*DYSF*	Ex13: NM_003494.4: c.1276G>A; p.(Gly426Arg) HEZ	2:71755523	PM3_strong, PM2, PP3, PP4_mod	Likely pathogenic
							Int44: NM_003494.4: c.4887-2A>G HEZ	2:71891396	PSV1, PP4_mod, PM2, PM3_strong	Pathogenic
P23	Myo080	Dysferlinop.	Bx, MRI, lab, EcoK, Sp. EMG, Ab.	LGMD R2-Dysferlin related myopathy	NGS-DLE	*DYSF*	Ex27: NM_003494.4:c.2858dupT; p.(Phe954ValfsTer2) HEZ	2:71796997	PVS1, PM2, PM3, PM4, PP4_mod	Pathogenic
							Ex34: NM_003494.4:c.3770G>A; p.(Trp1257Ter) HEZ	2:71827899	PSV1, PP4_mod, PM2, PM3	Pathogenic
P24	Myo084	LGMD-R?	Bx, MRI, lab, EcoK, Sp. EMG, Ab	Unknown	DLE-NGS, CL-NGS, CNMDP	*Nonet*				
P25	Myo086	LGMD-R1	Bx, MRI, lab, EcoK, Sp. EMG, Ab	Dystrophinopathy (BMD)	DLE-NGS, CNMDP	*DMD*	Ex2: NM_004006.2:c.40_41del; p.(Glu14ArgfsTer17) HMZ	X:33038308-33038309	PSV1, PM2, PM4, PP4_mod, PP5	Pathogenic
P26	Myo088	LGMD-R2	Bx, MRI, lab, EcoK, Sp. EMG	LGMD R2-Dysferlin related myopathy	NGS-DLE	*DYSF*	Int44: NM_003494.4:c.4887-2A>G HEZ	2:71891396	PSV1, PP4_mod, PM2, PM3_strong	Pathogenic
							Ex26: NM_003494.4:c.2779delG; p.(Ala927LeufsTer21) HEZ	2:71795437	PVS1, , PP1 supp; PM3 strong, PM2, PP4_mod	Pathogenic
P27	Myo089	LGMW-CMS	Bx, MRI, lab, EcoK, Sp. EMG	LEMS. P/Q-Type Calcium Channel Ab	DLE-NGS, CNMDP	*TTN*	Ex343: NM_001267550.1: c.95195C>T; p.(Pro31732Leu) HEZ	2:179410768	PM2, PP3, PM1, PS4, PS3	Pathogenic
						CACNA1S	Ex.13: NM_000069.2:c.1847G>A; p.(Trp616Ter) HEZ	1:201044724	PVS1, PM2, PS4_sup	Pathogenic

Supplementary Table S1. Summary of the clinical and genetic screening of the Chilean series of patients with LGMW (Continued)										
ID	Local ID	Suspected Diagnosis	Other diagnostic tests	Definite Diagnosis	Screening Method	Affected Gen	Pathogenic or most probably potentially pathogenic variants found	Genomic Position (hg19)	ACMG codes	ACMG classification
P28	Myo090	LGMW	Bx, MRI, lab, EcoK, Sp. EMG, Ab	*VCP*-related myopathy	DLE-NGS, CL-NGS, CNMDP	*VCP*	Ex.6: NM_007126.3:c.648A>G; p.(Ile216Met) HEZ	9:35064211	PM2, PP3, PP4_mod.	VUS
P29	Myo091	Dysferlinop.	Bx, MRI, lab, EcoK, Sp. EMG	LGMDR2-Dysferlin related myopathy	NGS-DLE	*DYSF*	Int44: NM_003494.4:c.4887-2A>G HEZ	2:71891396	PSV1, PP4_mod, PM2, PM3_strong	Pathogenic
							Ex26: NM_003494.4:c.2779delG; p.(Ala927LeufsTer21) HEZ	2:71795437	PVS1, , PP1 supp; PM3 strong, PM2, PP4_mod	Pathogenic
P30	Myo092	LGMD?	Bx, MRI, lab, EcoK, Sp. EMG	*RYR1*-related myopathy	DLE-NGS, CNMDP	*RYR1*	Ex31: NM_000540.2: c.4455_4459dup; p.(Lys1487TfsTer16) HEZ	19:38969075_38969079	PVS1, PM2, PS2??, PM3_sup, PP4_mod	Pathogenic
							Ex39: NM_000540.2:c.6502G>A; p.(Val2168Met) HEZ	19:38985219	PM1, PM2, PP3, PP4, PM3_strong	Pathogenic
P31	Myo093	LGMD?	Bx, MRI, lab, EcoK, Sp. EMG	Unknown	CL-NGS	*None*				
P32	Myo094	Dysferlinop.	Bx, MRI, lab, EcoK, Sp. EMG	LGMDR2-Dysferlin related myopathy. DYSF absent in muscle biopsy	NGS-DLE	*DYSF*	5´UTR: NM_003494.3: c.-116delG HOZ	2:71681013	PM2, PP4_mod	VUS
P33	Myo095	hiCK, myalgia	Bx, lab, EcoK, Sp. EMG	Unknown. Normal dysferlin expression	NGS-DLE	*DYSF*	Ex53: NM_003494.3: c.5979dupA, p.(Glu1994ArgfsTer3) HEZ	2:71908163	PVS1, PM3 very strong, PM2, PP4_mod, PP1_sup.	Pathogenic
P34	Myo096	LGMD, dyspnea	Bx, MRI, lab, EcoK, Sp. EMG, DBS	LO Pompe Disease (former LGMD2V)	NGS-DLE	*GAA*	Int1: NM_000152.4: c.-32-13T>G HEZ	17:78078341	PVS1_strong, PM3_strong, PP4_mod, BS1	Pathogenic
							Ex18:NM_000152.4:c.2560C>T; p.(Arg854Ter) HEZ	17:78092070	PVS1, PM2, PM3	Pathogenic
P35	Myo097	EDMD	Bx, MRI, lab, EcoK, Sp. EMG	Laminopathy A/C (former LGMD1B)	CNMDP	*LMNA*	Ex7: NM_170707.3:c.1357C>T; p.(Arg453Trp) HEZ	1:156106204	PS3, PS4_Mod, PM1, PM2, PP4_mod, PP3	Pathogenic
P36	Myo099	hiCK, myalgia	Bx, MRI, lab, EcoK, Sp. EMG	McArdle's disease	CNMDP	*PYGM*	Ex1: NM_005609.2:c.148C>T, p.(Arg50Ter) HEZ	11:64527223	PVS1, PS3, PM3, PP1	Pathogenic
							Del Ex.15-16 HEZ: NM_005609.2: c.(1768+1_1769-1)_(1969+1_1970-1)del: p.(Arg590_Val657del)		PVS1_Strong, PP4_mod	Likely Pathogenic
P37	Myo100	LGMD-R1	Bx, MRI, lab, EcoK, Sp. EMG, SEG	LGMDR1-Calpain 3 related myopathy	CL-NGS	*CAPN3*	Ex1: NM_000070.3:c.107delG: p.(Gly35fsTer21) HEZ	15:42652105	PVS1, PM2, PM3, PP4_mod	Pathogenic
							Ex22: NM_000070.3:c.2362_2363delinsTCATCT; p.(Arg788SerfsTer14) HEZ	15:42703180-42703181	PVS1, PM3_very_strong, PM2, PP4_mod	Pathogenic
P38	Myo101	hiCK, myalgia	Bx, MRI, lab, EcoK, Sp. EMG	Congenital Paramyotonia	CNMDP	*SCN4A*	Ex13: NM_000334.4:c.2078T>C, ; p.(Ile693Thr) HEZ	17:62034820	PS3, PS4_Mod, PM2, PP1, PP3, PP4_mod	Pathogenic
P39	Myo102	Dysferlinop.	Bx, lab, EMG	LGMDR2-Dysferlin related myopathy	DLE-NGS	*DYSF*	Ex27: NM_003494.4:c.2858dup; p.(Phe954ValfsTer2) HEZ	2:71796997	PVS1, PM2, PM3, PM4, PP4_mod	Pathogenic
							Ex53: NM_003494.3: c.5979dup; p.(Glu1994ArgfsTer3) HEZ	2:71908163	PVS1, PM3_strong, PM2, PP4_mod, PP1_supp	Pathogenic
P40	Myo103	hiCK, myalgia	Bx, lab, EMG	Unknown	NGS-DLE	*None*				

Supplementary Table S1. Summary of the clinical and genetic screening of the Chilean series of patients with LGMW (Continued)										
ID	Local ID	Suspected Diagnosis	Other diagnostic tests	Definite Diagnosis	Screening Method	Affected Gen	Pathogenic or most probably potentially pathogenic variants found	Genomic Position (hg19)	ACMG codes	ACMG classification
P41	Myo103.1	hiCK, myalgia	Bx, lab, EMG	Unknown	NGS-DLE	*None*				
P42	Myo104	LGMDR, myositis	Bx, lab, EMG	Dysferlinopathy?	NGS-DLE	*DYSF*	Ex14: NM_003494.3:c.1186G>A, p.(Asp396Ala) HOZ	2:71755433	PM1, PM2, PP3	VUS
P43	Myo105	LGMD-R?	Bx, lab, EMG	Unknown	NGS-DLE	*None*				
P44	Myo106	LGMD-R?	Bx, lab, EcoK, Sp. EMG	Dystrophinopathy? DYS reduced in muscle biopsy	DLE-NGS, CNMDP	*None*				
P45	Myo107	LGMD-R?	Bx, lab, EcoK, Sp. EMG	LGMDR4 β-Sarcoglycan related myopathy	DLE-NGS	*SGCB*	Int4: NM_000232.4:c.621+1G>A HOZ	4:52894895	PVS1, PM2, PP4_mod	Pathogenic
P46	Myo109	LGMD-R?	Bx, MRI, lab, EcoK, Sp. EMG	LGMDR1-Calpain 3 related myopathy	CL-NGS	*CAPN3*	Ex19: NM_000070.2:c.2105C>T; p.(Ala702Val) HEZ	15:42702183	PM2, PP3, PP4_mod, PM3_very_strong	Pathogenic
							Ex22: NM_000070.3:c.2362_2363delinsTCATCT; p.(Arg788SerfsTer14) HEZ	15:42703180-42703181	PVS1, PM3_very_strong, PM2, PP4_mod	Pathogenic
P47	Myo111	LGMW	Bx, MRI, lab, EcoK, Sp. EMG, SEG	*MTM1*-Related Congenital Myopathy	CNMDP	*MTM1*	Int11: NM_000252.2:c.1261-10A>G; HEZ	X:149828127	PP3, PM2, PS4, PP4_mod	Pathogenic
P48	Myo115	LGMD, myalgia	Bx, MRI, lab, EcoK, Sp. EMG, Ab.	Unknown	CL-NGS, CNMDP	*None*				
P49	Myo116	LGMD, myalgia	Bx, MRI, lab, EcoK, Sp. EMG	LGMDR12-Anoctamin 5 related myopathy	CL-NGS	*ANO5*	Ex19: NM_213599.3:c.2201T>C; p.(Leu734Pro) HOZ	11:22294501	PM2, PM3_sup, PP3, PP4_mod??	Likely pathogenic
							Ex5: NM_213599.3:c.191dupA; p.(Asn64LysfsTer15) HEZ	11:22242646	PM2, PM3, PP3, PP4_mod??	Likely Pathogenic
P50	Myo117	LGMD	Bx, MRI, lab, EcoK, Sp. EMG, Ab.	IMNM Anti-HMGCR	DLE-NGS, CNMDP	*None*				
P51	Myo118	Emery Dreifuss	Bx, lab,	XL-Emery Dreyfus muscular dystrophy	NGS-DLE, CNMDP	*EMD*	Ex6: NM_000117.3:c.619del; p.(Arg207GlyfsTer30) HMZ	X:153609411	PVS1_Strong, PS4_mod, PM2, PP4_mod	Pathogenic
P52	Myo119	LGMW	Bx, MRI, lab, EcoK, Sp. EMG	Unknown	CNMDP	*None*				
P53	Myo120	LGMW	Bx, lab, EcoK, Sp. EMG	Dystrophinopathy? Reduced DYS expression in muscle biopsy	CNMDP[(4b)]	*None*				
P54	Myo121	LGMW	Bx, MRI, lab, EcoK, Sp. EMG,	LGMDR3 α-Sarcoglycan related myopathy	CL-NGS	*SGCA*	Ex3: NM_000023.3:c.229C>T, p.(Arg77Cys) HEZ	17:48245014	PP3, PP4_mod, PM3_very strong	Pathogenic
							Ex6: NM_000023.3:c.746T>C, p.(Leu249Pro) HEZ	17:48246614	PM2, PP3, PP4_mod, PM3, PP1_strong	Pathogenic
P55	Myo126	LGMW, dyspnea	Bx, Lab, EMG, EcoK, Sp.	Unknown	NGS-DLE, CNMDP	*None*				
P56	Myo128	LGMD-R1	Bx, MRI, lab, EcoK, Sp. EMG	LGMDR22-COL6A3 related myopathy	CL-NGS, CNMDP	*COL6A3*	Ex10: NM_004369.4:c.4899del, p.(Glu1634ArgfsTer32) HOZ	2:237368564	PVS1, PM2, PM3_sup, PP4_mod	Pathogenic

Supplementary Table S1. Summary of the clinical and genetic screening of the Chilean series of patients with LGMW (Continued)

ID	Local ID	Suspected Diagnosis	Other diagnostic tests	Definite Diagnosis	Screening Method	Affected Gen	Pathogenic or most probably potentially pathogenic variants found	Genomic Position (hg19)	ACMG codes	ACMG classification
P57	Myo131	LGMW	Bx, MRI, lab, EcoK, Sp. EMG	LGMDR10-Titin related myopathy?	CNMDP	*TTN*	Ex326:NM_001267550:c.70897dup; p.(Leu23633ProfsTer17) HEZ	2:179439962	PVS1, PM2, PS4_sup	Pathogenic
P58	Myo132	LGMW	Bx, MRI, lab, EcoK, Sp. EMG	Dystrophinopathy (BMD)	CNMDP	*DMD*	Ex2: NM_004006.2:c.40_41del; p.(Glu14ArgfsTer17) HMZ	X:33038308-33038309	PSV1, PS4_sup, PM2	Pathogenic
P59	Myo137	Dysferlinop.	Bx, MRI, lab, EcoK, Sp. EMG	LGMDR2-Dysferlin related myopathy	CL-NGS	*DYSF*	Ex42: NM_003494.3:c.4491G>T, p.(Lys1497Asn) HEZ	2:71871175	PM2, PP4_mod, PM3_sup	VUS
							Ex53: NM_003494.3: c.5979dup, p.(Glu1994ArgfsTer3) HEZ	2:71908163	PVS1, PM3_strong, PM2, PP4_mod, PP1_supp	Pathogenic
P60	Myo139	LGMW	Bx, MRI, lab, EcoK, Sp. EMG	Dystrophinopathy (BMD)	CNMDP	*DMD*	Ex. 2-45: NM_004006.2: c.(31+1_32-1)_(6614+1_6615-1)del ; p.(Tyr11CysfsTer2), out of frame HMZ		PSV1, PM1, PP4	Pathogenic
P61	Myo140	Dysferlinop.	Bx, MRI, lab, EcoK, Sp. EMG	LGMDR2-Dysferlin related myopathy. DYSF absent in muscle biopsy	CNMDP	DYSF	Ex50: NM_003494.3:c.5594delG, p.(Gly1865AlafsTer101) HEZ	2:71896803	PVS1, PM3_strong, PM2, PP4_mod, PP1_mod	Pathogenic
P62	Myo141	LGMD-R1	Bx, MRI, lab, EcoK, Sp. EMG	*RYR1*-related myopathy?	CNMDP	*RYR1*	Int14: NM_000540.2: c.1577-5C>G HEZ	19:38946086	PM2, PP3, PM6	VUS
P63	Myo142	LGMW, distal	Bx, MRI, lab, EcoK, Sp. EMG	*RYR1*-related myopathy	CNMDP	*RYR1*	Ex98: NM_000540.2: c.14209C>T; p.(Arg4737Trp) HEZ	19:39068594	PS4_mod, PM1, PM2, PP3, PP1_mod	Likely Pathogenic
P64	Myo142.1	LGMW, distal	Lab, EMG	*RYR1*-related myopathy	CNMDP	*RYR1*	Ex98: NM_000540.2: c.14209C>T; p.(Arg4737Trp) HEZ	19:39068594	PS4_mod, PM1, PM2, PP3, PP1_mod	Likely pathogenic
P65	Myo143	LGMW	Bx, MRI, lab, EcoK, Sp. EMG	*SYNE2*-related myopathy?	CNMDP	*SYNE2*	Ex34: NM_182914.2: c.4933C>G; p.(Leu1645Val), HEZ	14:64484358	PM2, PP4	VUS
P66	Myo144	LGMD, dyspnea	Bx, MRI, lab, EcoK, Sp. EMG	*TPM3*-related congenital myopathy. CFTD and Cap Disease	CNMDP	*TPM3*	Ex8: NM_152263.4:c.709G>A, p.(Glu237Lys) HTZ	1:154142942	PS4_sup, PM2, PP3, PP4_mod	Likely Pathogenic
P67	Myo145	Dysferlinop.	Lab, EcoK, Sp. EMG	LGMDR2-Dysferlin related myopathy	CNMDP	*DYSF*	Ex53: NM_003494.3: c.5979dup; p.(Glu1994ArgfsTer3) HOZ	2:71908163	PVS1, PM3_strong, PM2, PP4_mod, PP1_supp	Pathogenic
P68	Myo146	LGMD	Lab, EcoK, Sp. EMG	LGMDD5-COL6A1-related myopathy	CNMDP	*COL6A1*	Int14: NC_000021.9: c.1056+5G>A HEZ	21:47410745	PS2, PS4_mod, PM4, PM2, PP3, PP4	Pathogenic
P69	Dysf015 H1	Dysferlinop.	Bx, lab, EcoK, Sp.	LGMDR2-Dysferlin related myopathy	NGS-DLE	*DYSF*	Ex27: NM_003494.4:c.2858dup ; p.(Phe954ValfsTer2) HOZ	2:71796997	PVS1, PM2, PM3, PM4, PP4	Pathogenic
P70	Myo135	LGMD-R1	Bx, MRI, lab, EcoK, Sp. EMG	SMN1 / SMA3	CL-NGS, MLPA(5)	*SMN1*	NM_000344.3: SMN1 0 copies		PVS1, PP4_mod	Pathogenic
P71	Myo138	LGMW	MRI, lab, EcoK, Sp. EMG	SMN1 / SMA3	CL-NGS, MLPA	*SMN1*	NM_000344.3:SMN1 0 copies; NM_017411.3:SMN2 3 copies		PVS1, PP4_mod	Pathogenic
P72	Myo148	LGMW, distal	Bx, EMG	desmin-related myopathy (former LGMD1E)	CNMDP	*DES*	Ex 6: NM_001927.4: c.1049G>C, p.(Arg350Pro) HEZ	2:220286087	PS3, PS4_mod, PM2, PP3, PP4_mod	Pathogenic

Supplementary Table S1. Summary of the clinical and genetic screening of the Chilean series of patients with LGMW (Continued)										
ID	Local ID	Suspected Diagnosis	Other diagnostic tests	Definite Diagnosis	Screening Method	Affected Gen	Pathogenic or most probably potentially pathogenic variants found	Genomic Position (hg19)	ACMG codes	ACMG classification
P73	Myo149	Dysferlinop.	EMG	LGMDR2-Dysferlin related myopathy	CNMDP	*DYSF*	Ex26: NM_003494.4:c.2779del ; p.(Ala927LeufsTer21) HEZ	2:71795437	PVS1, PM3_strong, PM2, PP1, PP4_mod	Pathogenic
							Ex53: NM_003494.3: c.5979dupA; p.(Glu1994ArgfsTer3) HEZ	2:71908163	PVS1, PM3_strong, PM2, PP4_mod, PP1	Pathogenic
						GFPT1	Int8: NM_001244710.1: c.686-2A>G HEZ	2:69581446	PSV1, PS4_mod, PM2	Pathogenic
P74	Myo150	Dysferlinop.	EMG	LGMDR2-Dysferlin related myopathy	CNMDP	*DYSF*	Ex53: NM_003494.3: c.5979dup ; p.(Glu1994ArgfsTer3) HOZ	2:71908163	PVS1, PM2, PM3_strong, PP4_mod, PP1	Pathogenic
P75	Myo151	LGMW	Bx	Dystrophinopathy (BMD)	CNMDP	*DMD*	Ex.10-22: NM_004006.2:c.(960+1_961-1)_(2949+1_2950-1)del; p.(His320_Ala983del) In frame deletion HMZ		PSV1_strong, PM1, PM2, PP4_mod	Pathogenic
P76	Myo152	LGMW	Bx, MRI, Sp., EMG	*VCP*-related myopathy	CNMDP	*VCP*	Ex5: NM_007126.3:c.463C>T ; p.(Arg155Cys) HEZ	9:35065361	PM1, PM2, PM5, PP3, PP4_mod	Likely Pathogenic
P77	Myo153	LGMW, hiCK	Bx, EMG	McArdle's disease	CNMDP	*PYGM*	Ex1 : NM_005609.2 : c.148C>T ; p.(Arg50Ter) HOZ	11:64527223	PVS1, PS3, PM3_Strong, PP4, BS1	Pathogenic
P78	Myo154	LGMW, dyspnea	Bx, MRI, EMG, Sp. EcoK	LO-Pompe disease (Former LGMD2V)	CNMDP	*GAA*	Int12: NM_000152.4: c.1755-1G>A HEZ	17:78086376	PVS1, PM2, PM3, PP4	Pathogenic
							Ex11: NM_000152.4:c.1559A>G; p.(Asn520Ser) HEZ	17:78084747	PM1, PM2, PP3, PP4_mod	Likely Pathogenic
P79	Myo155	Dysferlinop.	EMG	LGMDR2-Dysferlin related myopathy	CNMDP	*DYSF*	Ex26: NM_003494.4:c.2779del; p.(Ala927LeufsTer21) HEZ	2:71795437	PVS1, PM2, PM3_strong, PP1, PP4_mod	Pathogenic
							Ex53: NM_003494.3: c.5979dup ; p.(Glu1994ArgfsTer3) HEZ	2:71908163	PVS1, PM2, PM3_strong, PP1, PP4_mod	Pathogenic
						GFPT1	Int8: NM_001244710.1: c.686-2A>G HEZ	2:69581446	PSV1, PS4_mod, PM2	Pathogenic
P80	Myo156	Dysferlinop.	EMG	LGMDR2-Dysferlin related myopathy	CNMDP	*DYSF*	Ex26: NM_003494.3:c.2779del; p.(Ala927LeufsTer21) HEZ	2:71795437	PVS1, PM2, PM3_strong, PP1, PP4_mod	Pathogenic
							Ex40: NM_003494.3:c.4390G>T, p.(Glu1464Ter) HEZ	2:71840520	PVS1, PM2, PM3, PP4_mod	Pathogenic
P81	Myo157	LGMW	MRI, Bx,	LGMDR5 γ-Sarcoglycan related myopathy. SGCG absent in muscle biopsy	CNMDP	*SGCG*	Ex8: NM_000231.2:c.817T>A p.(Tyr273Asn) HTZ	13:23898621	PM2, PP3, PP4_mod	VUS
P82	Myo158	Dysferlinop.	EMG, Bx	LGMDR2-dysferlin related myopathy. DYSF absent in muscle biopsy	CNMDP	*DYSF*	Ex27: NM_003494.4:c.2858dup; p.(Phe954ValfsTer2) HEZ	2:71796997	PVS1, PM2, PM3, PP4_mod	Pathogenic

Supplementary Table S1 References: hi-CK, hyperckemia; LGMD, limb girdle muscular dystrophy; LGMDW, limb girdle muscular weakness; CMS, congenital myasthenic syndrome; EDMD, Emery Dreifuss Muscular Dystrophy phenotype, Bx, muscle biopsy; MRI, muscle MR imaging; Lab: CK levels; EMG, electrophysiology; EcoK; Echocardiogram; Sp, Spirometry; SEG, segregation study completed by Sanger; Ab, serology for anti-myositis specific, associated antibodies and anti-HMGCR antibodies. IMNM, immune mediated necrotizing myopathy; DBS, dry blood spot test for Pompe's disease; HOZ, homozygous; HEZ, heterozygous; HMZ, hemizygous. All mutations were confirmed by Sanger. NGS methods used **(1) MyoPanel2**, 306 genes panel (Marseille, France); **(2) DLE-NGS (Sanofi-Genzyme)**, 10 genes panel, 9 most frequent recessive LGMD and Pompe disease (DLE Labs, Sao Paulo, Brazil); **(3) CL-NGS**, locally designed 15 LGMD-genes panel; **(4a) CNMDP-Invitae**^{TM}. 123-gene comprehensive neuromuscular disorders panel or **(4b)** 137-gene comprehensive neuromuscular disorders panel was used only in case P53-Myo120 (California, USA). **(6)** MLPA for SMN1 and SMN2 in P70-Myo135 and P71-Myo138 (See Supplementary File 1)
